# Generalized gene co-expression analysis via subspace clustering using low-rank representation

**DOI:** 10.1186/s12859-019-2733-5

**Published:** 2019-05-01

**Authors:** Tongxin Wang, Jie Zhang, Kun Huang

**Affiliations:** 1 Department of Computer Science, Indiana University Bloomington, Bloomington, 47408 IN USA; 2 Department of Medical and Molecular Genetics, Indiana University School of Medicine, Indianapolis, 46202 IN USA; 3 Department of Medicine, Indiana University School of Medicine, Indianapolis, 46202 IN USA; 4 Regenstrief Institute, Indianapolis, 46202 IN USA

**Keywords:** Gene co-expression network analysis, Subspace clustering, Low-rank representation

## Abstract

**Background:**

Gene Co-expression Network Analysis (GCNA) helps identify gene modules with potential biological functions and has become a popular method in bioinformatics and biomedical research. However, most current GCNA algorithms use correlation to build gene co-expression networks and identify modules with highly correlated genes. There is a need to look beyond correlation and identify gene modules using other similarity measures for finding novel biologically meaningful modules.

**Results:**

We propose a new generalized gene co-expression analysis algorithm via subspace clustering that can identify biologically meaningful gene co-expression modules with genes that are not all highly correlated. We use low-rank representation to construct gene co-expression networks and local maximal quasi-clique merger to identify gene co-expression modules. We applied our method on three large microarray datasets and a single-cell RNA sequencing dataset. We demonstrate that our method can identify gene modules with different biological functions than current GCNA methods and find gene modules with prognostic values.

**Conclusions:**

The presented method takes advantage of subspace clustering to generate gene co-expression networks rather than using correlation as the similarity measure between genes. Our generalized GCNA method can provide new insights from gene expression datasets and serve as a complement to current GCNA algorithms.

## Background

Gene Co-expression Network Analysis (GCNA) is a popular method in bioinformatics and biomedical research to construct gene co-expression networks and detect co-expressed genes. It has been widely utilized in many applications, such as gene function prediction [1–3], disease biomarker discovery [4, 5], protein-protein interaction (PPI) inference [6] and genetic variants detection in cancers [7, 8].

Many GCNA algorithms have been developed to identify modules of co-expressed genes [3, 9–13]. One of the widely used GCNA tools is the WGCNA package developed by Horvath’s group [10], which finds modules of highly correlated genes in weighted gene co-expression networks. Local maximal Quasi-Clique Merger (lmQCM) [13] is another weighted GCNA algorithm that allows overlap between gene modules, which is consistent with the fact that the same gene may participate in multiple biological processes.

Mathematically, given the expression matrix **G** for *K* genes and *N* samples

$$\mathbf{G}=\left[\begin{array}{c} \mathbf{g_{1}}\\ \mathbf{g_{2}}\\...\\ \mathbf{g_{K}} \end{array}\right] \in \Re^{K\times N} $$

with *g*_*i*_^*T*^∈ℜ^*N*^ being the expression profile for gene *i* (*i*∈{1,2,...,*K*}), the geneco-expression network can be represented by a matrix **W**∈ℜ^*K*×*K*^, where entry *W*_*i*,*j*_ represents the co-expression similarity between expression profiles of a pair of genes *g*_*i*_ and *g*_*j*_ (*i*,*j*∈{1,2,...,*K*}). Commonly, this similarity is measured using correlation coefficients, with Pearson Correlation Coefficient (PCC) or Spearman Correlation Coefficient (SCC) being the most widely used ones [3, 9, 13]. When using PCC to measure co-expression similarity, we obtain gene modules with linearly correlated gene expression profiles. In this case, expression profiles of gene *i* and *j* (*i*≠*j*) from the same co-expression module differ by a scale and a shift, i.e.,

1 $$ \mathbf{g_{i}} = \alpha_{j}\mathbf{g_{j}}+\beta_{j}\cdot \mathbf{1}_{1\times N}   $$

where *α*_*j*_ and *β*_*j*_ are scalars. It can be easily shown that the rank of such expression matrix for a co-expression module *G*_*c*_ is 2. In other words, expression profiles for genes in a co-expression module can be approximated as a subspace with dimensionality of two in a *N*-dimensional space. In addition, if we designate

$$\bar{\mathbf{g_{i}}}=\mathbf{g_{i}}-\bar{g_{i}}\cdot \mathbf{1}_{1\times N} $$

as the centralized version of *g*_*i*_, where $\bar {g_{i}}$ is the mean of entries in *g*_*i*_, then $\bar {\mathbf {g_{i}}}$s from the same co-expression module can be approximated by a 1-dimensional subspace.

However, apart from grouping genes that are linearly correlated into modules, simple relationships such as

2 $$ \mathbf{g_{i}} = \alpha_{j}\mathbf{g_{j}}+\alpha_{k}\mathbf{g_{k}}+\alpha_{m}\mathbf{g_{m}}   $$

where *g*_*j*_,*g*_*k*_ and *g*_*m*_ are linearly independent and *α*_*j*_,*α*_*k*_,*α*_*m*_≠0, cannot be captured using the traditional PCC based co-expression formulation. In this case, expression profiles for gene *i*, *j*, *k* and *m* cannot be approximated using a 2-dimensional subspace (in this example, they form a 3-dimensional subspace). However, it can be conceived that in biology, such coordinated gene activities may play important roles in complex processes and pathways. Therefore, there is a need to generalize the co-expression formulation to accommodate relationships between genes beyond pairwise relationships.

Discovering gene modules with such coherent relationships implies detecting low-dimensional subspaces in a higher dimensional space. *Subspace clustering* [14] is a research field in signal processing and machine learning for such purpose. The goal of subspace clustering is separating data according to their underlying subspaces, which could have different dimensionalities that are larger than one. Subspace clustering has found numerous applications in image processing and computer vision [15–18], as well as in bioinformatics [19–21].

One of the popular approaches for subspace clustering is Sparse Subspace Clustering (SSC) [22]. SSC is based on the affinity matrix defined by the sparsest representation produced by *l*_1_-minimization and subspace segmentation using spectral clustering. However, SSC may not be able to capture the global structure of the data accurately, which could affect the performance of the algorithm when data is highly corrupted. However, large biomedical datasets often contain large amount of noise and outliers. In addition, spectral clustering assigns every data point to a certain cluster, which can potentially bias the clustering structure.

Therefore, in this paper, we propose to use Low-Rank Representation (LRR) approach to construct gene co-expression networks from high-throughput gene expression data and use lmQCM to further group genes from the same subspace into gene modules. The lmQCM algorithm is developed by us as an extension of the QCM algorithm. It is a greedy algorithm for identifying highly connected modules in a large network with high efficiency. In addition, it allows overlap between clusters, which fits well with the notion that genes can participate in different functions and pathways.

Comparing to traditional GCNA algorithms based on correlation, our method provides a generalized formulation and can be applied to identify gene modules with expression matrices of higher intrinsic dimensionalities. This will help to discover new biological relationships, functions and pathways. Our method can also serve as a complement to current GCNA algorithms. Moreover, since LRR finds the lowest rank representation of all data jointly [18] and the corruption of data will largely increase the rank, LRR is robust to noise and outliers, making it suitable for analyzing high-throughput gene expression data. The contribution of this paper is a generalized gene co-expression network mining approach that is based on subspace clustering and demonstration of the effectiveness of this approach using real biomedical data.

## Methods

### Subspace clustering of gene expression data using LRR and lmQCM

Consider a data matrix **X**∈ℜ^*D*×*N*^ (each column is a sample) where each sample can be represented by a linear combination of columns in a dictionary **A**∈ℜ^*D*×*M*^.

3 $$ \mathbf{X} = \mathbf{A}\mathbf{Z} \\   $$

**Z**∈ℜ^*M*×*N*^ is a matrix with the *i*-th column being the representation of the *i*-th column in **X**. Introduced by Liu et al., LRR [18] uses low rankness of a matrix to capture the structure of the data and looks for a representation **Z** of data **X** by solving the following problem.

4 $$ \begin{aligned} & \underset{\mathbf{Z}}{\text{min}} & & \text{rank}(\mathbf{Z}) \\ & \text{s.t.} & & \mathbf{X} = \mathbf{A}\mathbf{Z} \\ \end{aligned}   $$

However, due to the discrete nature of the rank function, Problem (4) is difficult to solve. Instead, the following convex optimization problem is suggested as a surrogate for Problem (4) by matrix completion methods,

5 $$ \begin{aligned} & \underset{\mathbf{Z}}{\text{min}} & & \left\lVert{\mathbf{Z}}\right\rVert_{*} \\ & \text{s.t.} & & \mathbf{X} = \mathbf{A}\mathbf{Z} \\ \end{aligned}   $$

where ‖·‖_∗_ is the nuclear norm of a matrix.

In the case of gene expression data, we represent a dataset with *K* genes and *N* samples using a matrix **G** of size *K*×*N*. In order to cluster genes into their respective subspaces, we need to compute an affinity matrix encoding the pairwise similarities between genes by using the gene expression matrix itself as the dictionary [18]. We use **G**^*T*^ to substitute **X** in Problem (5) and Problem (5) becomes:

6 $$ \begin{aligned} & \underset{\mathbf{Z}}{\text{min}} & & \left\lVert{\mathbf{Z}}\right\rVert_{*} \\ & \text{s.t.} & & \mathbf{G}^{T} = \mathbf{G}^{T}\mathbf{Z} \\ \end{aligned}   $$

In reality, gene expression data often contains noise that could not be neglected. To take noise into account, we could modify the objective of Problem (6) into

7 $$ \begin{aligned} & \underset{\mathbf{Z},\mathbf{E}}{\text{min}} & & \left\lVert{\mathbf{Z}}\right\rVert_{*} + \lambda\left\lVert{\mathbf{E}}\right\rVert_{2,1} \\ & \text{s.t.} & & \mathbf{G}^{T} = \mathbf{G}^{T}\mathbf{Z} + \mathbf{E} \\ \end{aligned}   $$

where *λ*>0 is used to balance the effect of low rankness and noise. Problem (7) could be solved by solving an Augmented Lagrange Multiplier (ALM) problem using inexact ALM algorithms. After solving Problem (7), we define the adjacency matrix **W** of a weighted, undirected graph between genes based on the “lowest-rank representation" **Z**^∗^. The weight between gene *i* and *j*, *W*_*ij*_, is computed by $|Z^{*}_{ij}| + |Z^{*}_{ji}|$. As in [17], to deal with different norms of gene expression levels in **G** and ensure that the largest weights for all the genes are of the same scale, we also normalize the columns of **Z**^∗^ as *z*_*i*_=*z*_*i*_/‖*z*_*i*_‖_*∞*_, where *z*_*i*_ is the *i*-th column of **Z**^∗^.

Once we obtain the weighted network of genes, we need to cluster genes into their respective subspaces and perform further analysis on gene modules. Instead of using normalized cuts in [18] or spectral clustering in [17], we apply a recently developed weighted network mining algorithm called lmQCM [13]. Unlike normalized cuts or spectral clustering, which partitions genes into disjoint sets and does not allow overlaps between clusters, lmQCM is a greedy approach that allows genes to be shared among multiple clusters or not included in any cluster. This is consistent with subspace clustering problems where two subspace clusters can share some common genes or some genes may not belong to any subspace cluster [23]. Also, genes can participate in multiple biological processes, which could be represented by different clusters. Another advantage of lmQCM is that it can find gene co-expression modules potentially associated with Copy Number Variations (CNVs) in cancer development [13]. The lmQCM algorithm has four parameters *γ*, *α*, *t* and *β*. *γ* determines if a new module can be initiated by setting the weight threshold for the first edge of the module, and has the largest influence on the results. We use the default setting of *α*=1, *t*=1, *β*=0.4 in this paper and tune *γ* empirically.

Our method combines the strength of LRR and lmQCM, which we outline in Algorithm 1.


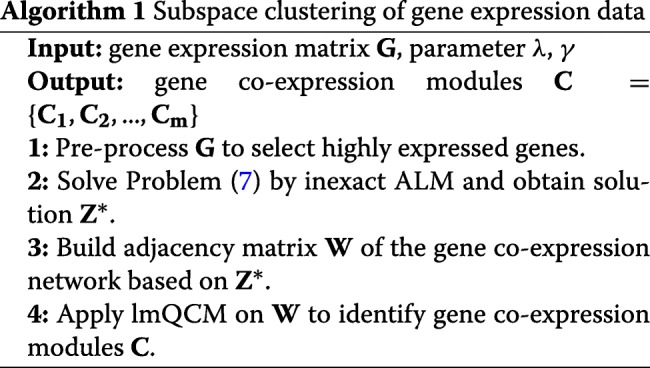


There are two parameters we need to choose in our method: *λ* for LRR and *γ* for lmQCM. In order to choose a set of parameters that is able to produce robust results, we randomly partition the dataset into 10 folds with the same size. Each time we use 9 folds to generate gene modules and we can get 10 sets of gene modules. We use *g*_10_ to denote the number of genes that appear in all 10 sets of gene modules and use *g*_1_ to denote the number of genes that appear in at least one set of gene modules. The value of *g*_10_/*g*_1_ can indicate the stability of the results under a certain set of parameters. We do not consider the parameters that can only identify less than nMdl_*thr*_ modules and choose the parameters that can produce the highest *g*_10_/*g*_1_ value.

### Functional enrichment analysis

Gene set functional enrichment analysis is a method to find biological annotations that are significant in a set of genes. In this study, we use annotations of Gene Ontology Biological Process (GO BP) terms from Gene Ontology Consortium and annotations of chromosome bands of protein coding genes from HGNC database. Hypergeometric probability density function from MATLAB Statistics and Machine Learning Toolbox with false discovery rate correction from MATLAB Bioinformatics Toolbox is used to determine the statistical significance. To provide meaningful results, we only perform functional enrichment analysis on gene modules with at least 10 genes and at most 500 genes.

For chromosome regions enriched by the identified gene modules, we are interested in if patients in other cohorts with the same disease have CNVs in these regions. We use OncoPrint visualization from the cBioPortal [24] website to investigate the Copy Number Alternations (CNAs) in selected genes. The Cancer Genome Atlas (TCGA) bladder cancer dataset (406 patients) was used to validate chromosome bands enrichment results of GSE31684. TCGA breast cancer dataset (996 patients) and METABRIC [25] breast cancer dataset (2051 patients) were used to validate chromosome bands enrichment results of GSE54002 and GSE102484.

### Gene expression data

Three large gene expression datasets were obtained from the NCBI Gene Expression Omnibus (GEO) and all datasets were generated using the Affymetrix Human Genome U133 Plus 2.0 Array Genechip with more than 54,000 probesets. Details of these datasets are summarized in Table 1.

**Table 1:** Summary of microarray datasets

Dataset	Disease	Number of samples	Platform
GSE31684	bladder cancer	93	GPL570
GSE54002	breast cancer	433	GPL570
GSE102484	breast cancer	683	GPL570

To reduce the size of affinity matrix between genes, pre-processing of each dataset is performed to select highly expressed genes. Firstly, only probesets with known associated genes are selected. If multiple probesets correspond to the same gene, only the probeset with the highest mean expression value is retained. Next, genes with low mean expression levels (bottom 20%) and low variance (bottom 10%) are removed using functions from MATLAB Bioinformatics Toolbox. Finally, we retain 10,000 genes with the highest mean expression levels for further analysis.

## Results

### LRR finds gene modules with different structures

Centralized Concordance Index (CCI) [26] is a linear algebraic based index for evaluating the concordance of gene co-expression modules from GCNA. A high CCI value suggests genes in a gene module are highly correlated, while a low CCI value suggests higher intrinsic dimensionality of expression profiles in a module. For example, when PCC between each pair of genes in a module is 1, CCI of this module is 1.

To compare our method with current correlation based GCNA algorithms, besides LRR, we also used PCC to generate the adjacency matrix of a gene co-expression network and applied lmQCM to identify gene co-expression modules. We calculated CCI of each gene module identified using PCC based method and our LRR based method. We observe that gene modules identified by LRR based method have significantly lower CCI values than those identified by PCC based method using Kolmogorov-Smirnov test (Table 2). This suggests that LRR, combined with lmQCM, can find gene co-expression modules from subspaces with higher dimensionality than current linear correlation based GCNA algorithms.

**Table 2:** Gene modules identified by LRR, PCC and WGCNA

Dataset	GSE31684	GSE54002	GSE102484
nMdl _LRR_	26	76	63
$\overline {\text {CCI}}_{\text {LRR}}$	0.487	0.471	0.457
nMdl _PCC_	97	100	81
$\overline {\text {CCI}}_{\text {PCC}}$	0.552	0.518	0.496
Kolmogorov-Smirnov test *P* value	2.43e-02	1.09e-04	9.22e-04
nMdl _WGCNA_	56	147	81

nMdl: number of gene modules identified

$\overline {\text {CCI}}$: mean of CCI values of gene modules identified

We also applied one of the state-of-the-art GCNA algorithms, WGCNA [10], for comparison. Since WGCNA partitions all the genes after pre-processing into disjoint sets rather than just identifies highly connected modules, CCI values of modules identified by WGCNA are not comparable with our lmQCM based results and we did not apply Kolmogorov-Smirnov tests on CCI values of identified gene modules between our method and WGCNA.

### Difference in CCI contributes to difference in enriched biological annotations

In the previous section, we have shown that gene modules identified by LRR based method have different structures from those identified by PCC based method, indicated by lower CCI values. In this section, we further demonstrate that such difference could lead to different enrichment results in biological annotations, such as GO BPs and chromosome bands. This suggests that our method is able to provide different biological insights than current correlation based GCNA algorithms and serve as a complement to current methods.

**Fig. 1:**
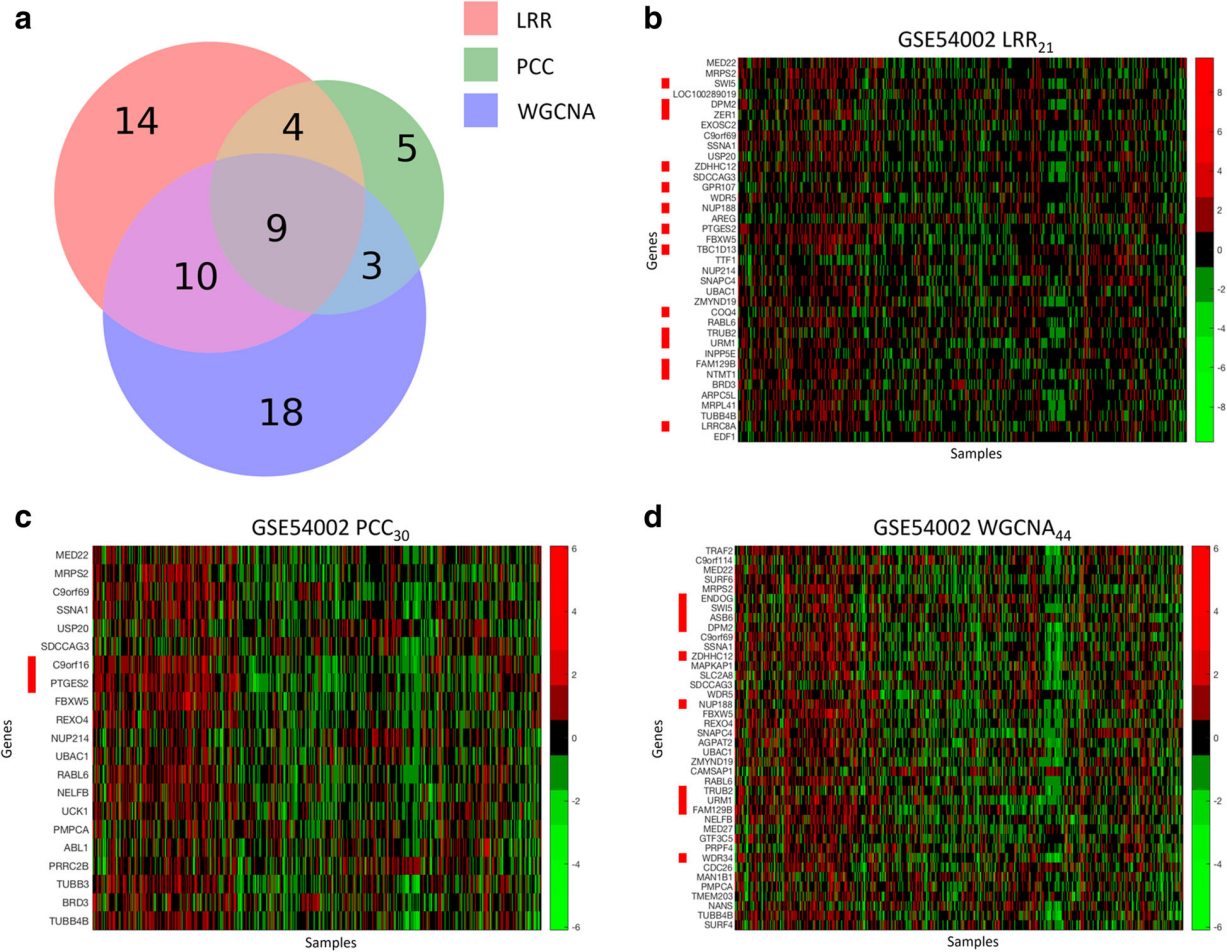
Comparison between LRR_21_, PCC_30_ and WGCNA_44_ in GSE54002. **a**. Venn diagram of genes in LRR_21_, PCC_30_ and WGCNA_44_; **b**-**d**. Heatmaps of expression profiles of LRR_21_, PCC_30_ and WGCNA_44_. Red bars beside gene symbols indicate genes on 9q34.11

We first compare three similar gene co-expression modules identified in GSE54002 dataset: LRR_21_ identified by our method, PCC_30_ identified by PCC based method and WGCNA_44_ identified by WGCNA. These three modules share a large fraction of the same genes and a Venn diagram of genes in these modules is shown in Fig. 1.

**Fig. 2:**
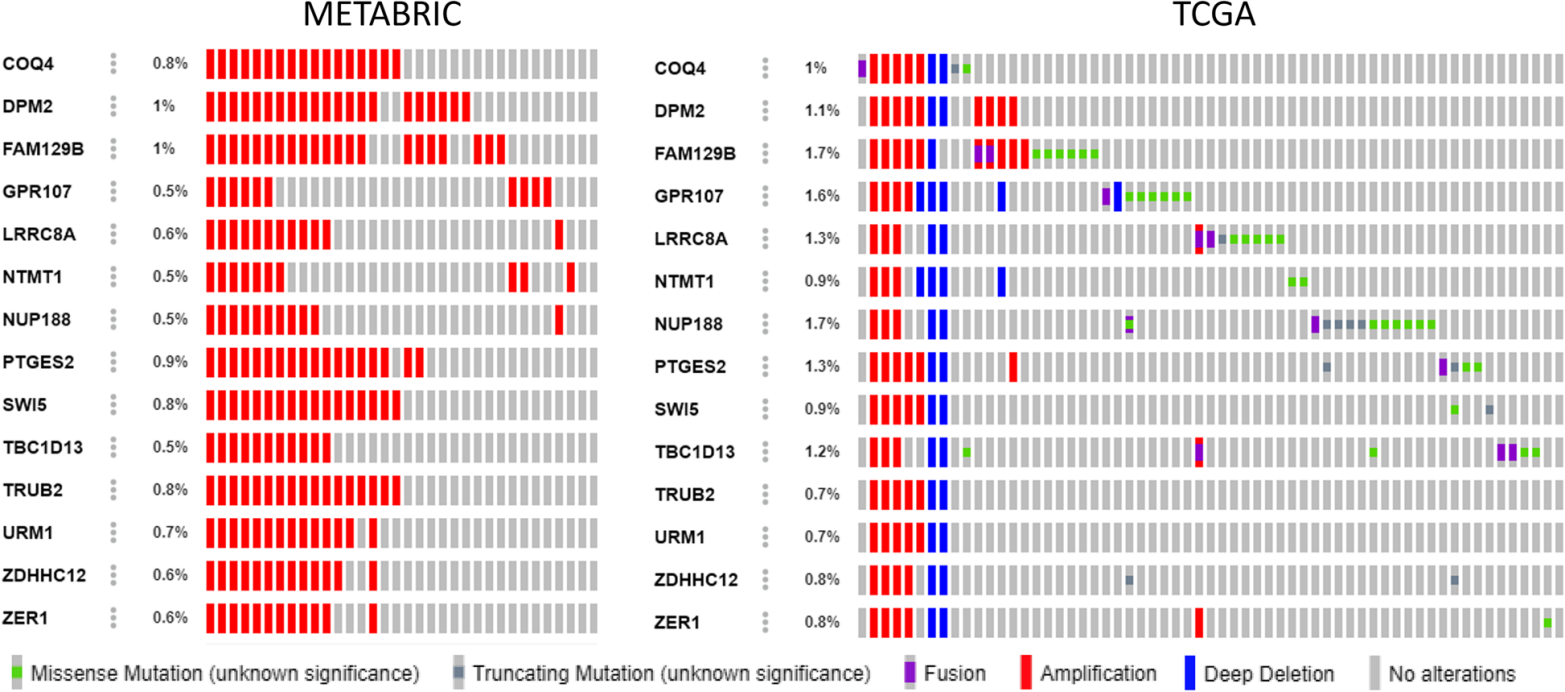
OncoPrint visualization of breast cancer patients with genetic mutations. Genes are from LRR_21_ which are also on 9q34.11

Figure 1 also shows heatmaps of gene expression profiles in these three modules. Expression levels of each gene have been standardized across different samples in order to show the correlation patterns between genes. We observe that PCC_30_ and WGCNA_44_ show a stronger correlation pattern between genes than LRR_21_, which is confirmed by higher CCI values (Table 3). However, some biological annotations are more enriched in LRR_21_ than in PCC_30_ or WGCNA_44_. For example, Table 3 shows chromosome band 9q34.11 is more enriched in LRR_21_ with *P* value =2.01×10^−26^. Figure 2 further shows that genes on the enriched 9q34.11 chromosome band in LRR_21_ share similar CNV patterns in the TCGA and METABRIC breast cancer patient cohorts.

**Table 3:** Summary of LRR_21_,PCC_30_ and WGCNA_44_ in GSE54002

Module	LRR_21_	PCC_30_	WGCNA_44_
number of genes	37	21	40
CCI	0.385	0.486	0.420
number of genes on 9q34.11	14	2	10
*P* value of enrichment analysis of 9q34.11	2.01e-26	2.38e-3	4.57e-17

**Fig. 3:**
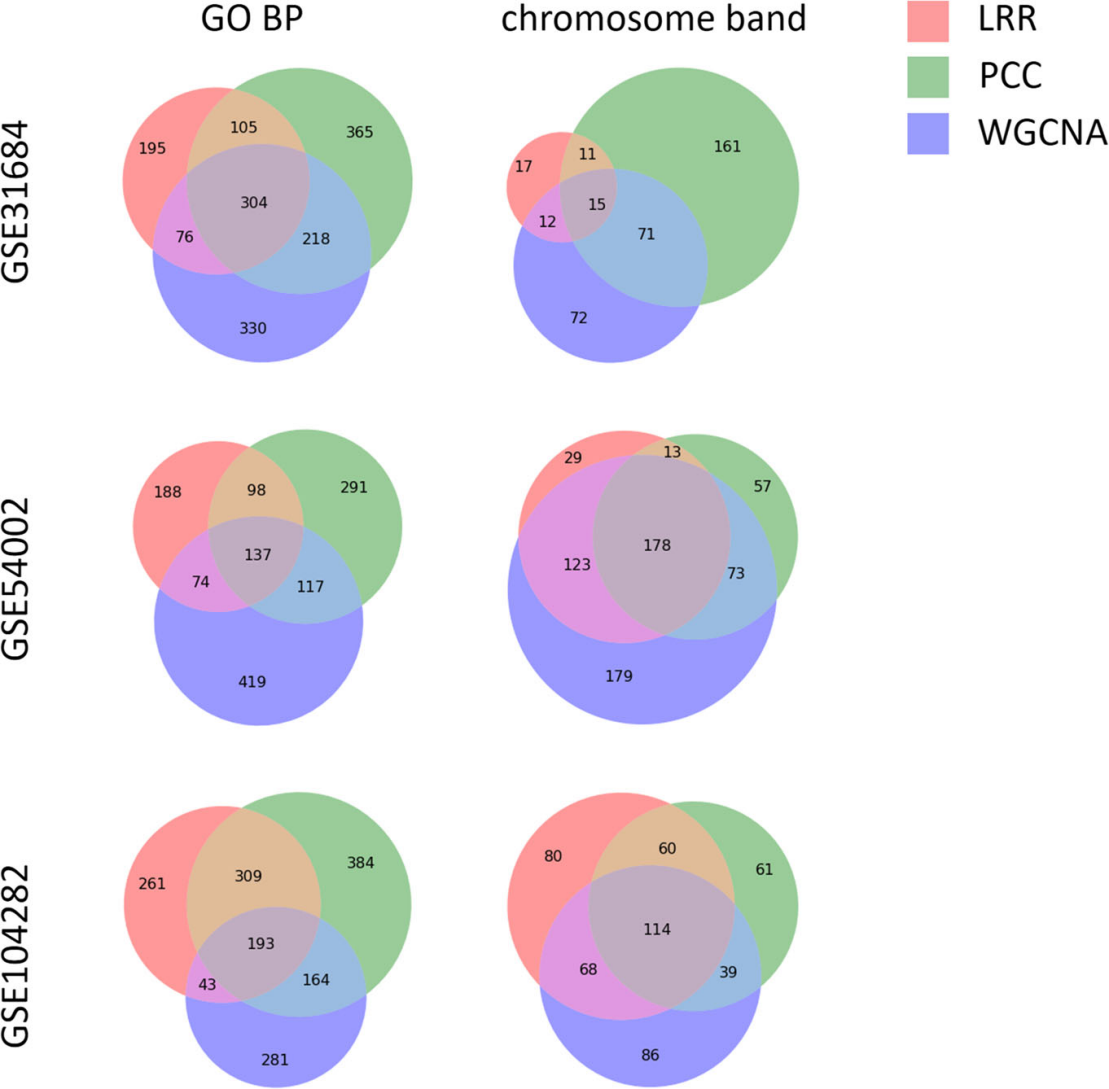
Number of enriched GO BPs and chromosome bands in microarray datasets. *P* value cutoff = 0.01

The aforementioned results suggest that by using LRR and allowing expression profiles of gene modules to have higher subspace dimensionality, we can identify biological annotations such as chromosome bands that are missed by correlation based GCNA algorithms. This may further lead to new discoveries of cancer-related structural mutations such as CNVs. Figure 3 provides the number of enriched GO BPs and chromosome bands using LRR, PCC based methods and WGCNA with a 0.01 *P* value cutoff. Our method not only produces results with substantial overlap between current GCNA methods in finding enriched biological annotations, but can also discover new related biological annotations. Such advantages give our method the potential to be combined with current GCNA methods to a get better understanding of gene expression data. Tables 4 and 5 list the most significant GO BPs and chromosome bands that are only enriched when using our method.

**Table 4:** The most significant GO BPs only enriched in our method

GO ID	Name	*P* value	Module ID
GSE31684			
GO:0043547	positive regulation of GTPase activity	3.59e-06	4
GO:0036336	dendritic cell migration	7.73e-06	4
GO:0043087	regulation of GTPase activity	2.89e-05	4
GO:0006929	substrate-dependent cell migration	9.66e-05	13
GO:0070232	regulation of T cell apoptotic process	1.07e-04	4
GSE54002			
GO:0000082	G1/S transition of mitotic cell cycle	1.27e-07	6
GO:0031109	microtubule polymerization or depolymerization	7.13e-07	6
GO:0000083	regulation of transcription involved in	8.48e-07	6
	G1/S transition of mitotic cell cycle		
GO:0007019	microtubule depolymerization	6.00e-06	6
GO:0051783	regulation of nuclear division	7.21e-06	6
GSE102484			
GO:0050766	positive regulation of phagocytosis	1.70e-06	1
GO:0006271	DNA strand elongation involved in DNA replication	1.95e-06	10
GO:0022616	DNA strand elongation	1.95e-06	10
GO:0050764	regulation of phagocytosis	3.18e-06	1
GO:0070374	positive regulation of ERK1 and ERK2 cascade	8.79e-06	1

*P* value cutoff = 0.01

**Table 5:** The most significant chromosome bands only enriched in our method

Chromosome band	*P* value	Module ID	CNVs validated in separate cohorts(TCGA, METABRIC)
GSE31684			
4q13.3	2.47e-06	13	Yes,NA
Yp11.2	8.25e-04	12	No,NA
Yq11.223	8.25e-04	12	No,NA
4q21.1	8.35e-04	4	Yes,NA
19p13.12	1.25e-03	5	No,NA
GSE54002			
11p15.1	1.92e-12	44	Yes,Yes
11q12.2	1.61e-06	19	Yes,Yes
19q13.31	1.03e-05	61	Yes,Yes
9q31.3	2.12e-05	26	No,Yes
9q22.33	2.28e-05	26	No,Yes
GSE102484			
8p11.23	3.97e-19	53	Yes,Yes
9q34.3	1.36e-09	14	Yes,Yes
15q22.31	4.21e-09	27	Yes,Yes
13q14.3	2.27e-08	18	Yes,Yes
7q36.3	4.12e-08	7	Yes,Yes

*P* value cutoff = 0.01

We also observe that for most chromosome bands that are only enriched in our method, genes on these chromosome bands in the corresponding co-expression modules often share similar CNV patterns among patients in the TCGA and METABRIC cohorts.

**Fig. 4:**
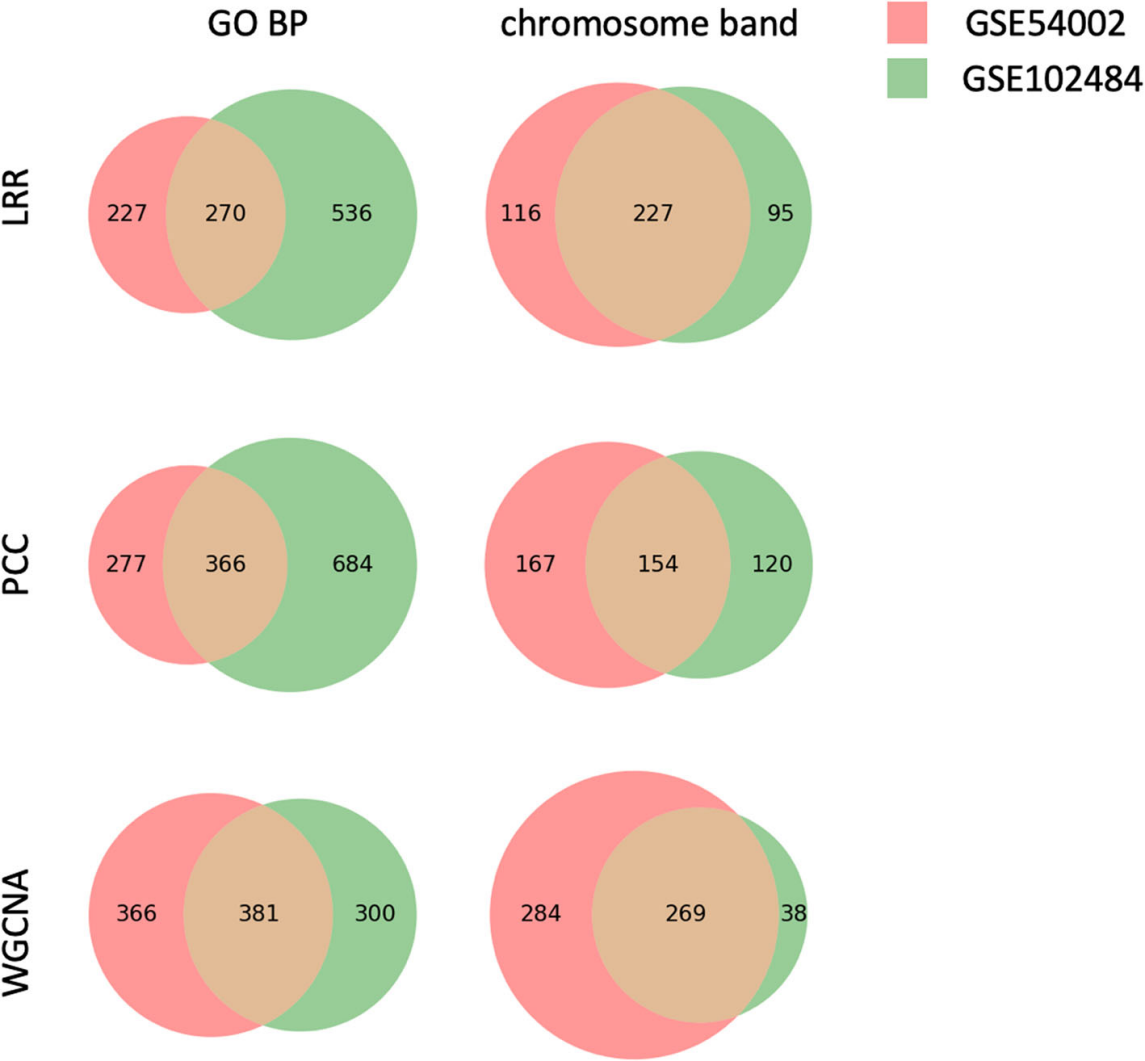
Number of overlapping enriched GO BPs and chromosome bands. GSE54002 and GSE102484 are two breast cancer datasets

We also compare overlap of enrichment analysis results between two breast cancer datasets, GSE54002 and GSE102484, using different GCNA methods, which is shown in Fig. 4. We observe that LRR and PCC based methods obtain similar fractions of overlap between two datasets, which indicates that our LRR based approach can achieve similar stability with the commonly used PCC based methods. WGCNA produced results with larger overlaps, which may be due to the fact that gene modules detected by WGCNA contains all the genes after pre-processing rather than just densely connected gene modules as in lmQCM. Since information of specific subtypes of breast cancer are unavailable for GSE54002 and GSE102484 on GEO, the difference of the enrichment analysis across these two datasets may come from the difference in breast cancer subtypes.

### Difference in CCI contributes to difference in PPI networks

To further validate our method, we use PINA2 platform [27] to verify whether LRR could identify gene co-expression modules that form PPI networks with different density. We use the Homo sapiens PPIs database from PINA2 website and map UniProtKB entries to gene symbols through UniProt website (uniprot.org). For a gene co-expression module with *n* genes and *n*_PPI_ PPIs within the module, we define PPI density of the module as 2*n*_PPI_/(*n*(*n*+1)) since the PINA2 database allows a protein to interact with itself. We found several cases where modules identified by LRR have lower CCI values and higher PPI densities than those identified by PCC or WGCNA. Some examples are shown in Table 6. This suggests that by identifying gene co-expression modules with higher intrinsic dimensionalities, we can find modules that are more densely connected in PPI networks, which indicates that genes in these modules share more similarities in biological functions.

**Table 6:** Examples of modules identified by our method with lower CCIs and higher PPI densities

Dataset	Module(nGenes)	nGenes _Overlap_	CCI	PPI density
GSE54002	LRR_5_(86)	16	0.457	3.81e-03
	PCC_14_(28)		0.498	0
GSE54002	LRR_5_(86)	54	0.457	3.81e-03
	WGCNA_18_(94)		0.467	2.38e-03
GSE54002	LRR_7_(78)	18	0.340	3.12e-03
	PCC_33_(20)		0.534	0
GSE54002	LRR_7_(78)	38	0.340	3.12e-03
	WGCNA_26_(77)		0.389	1.42e-03
GSE102484	LRR_17_(65)	15	0.291	2.46e-03
	PCC_24_(28)		0.400	0
GSE102484	LRR_17_(65)	28	0.291	2.46e-03
	WGCNA_23_(47)		0.382	1.03e-03
GSE102484	LRR_8_(96)	83	0.359	5.68e-03
	WGCNA_12_(115)		0.367	4.17e-03

nGenes: number of genes in the module nGenes _Overlap_: number of genes in both modules

### LRR helps identify prognostic gene modules

To determine whether a gene module has prognostic value, we use a lasso-regularized Cox proportional hazards model to calculate the risk index of each sample based on the expression profiles of the gene module. A leave-one-out cross validation strategy is used to validate our method, where each sample is used as a test sample and classified into a low-risk or a high-risk group. We then use Kaplan-Meier estimator and log-rank test to determine if these two groups have significantly distinct survival. We applied this method on GSE31684, using recurrence free survival months as survival time and recurrence/dod events as censoring information. We removed samples with survival time less than one month from analysis.

**Fig. 5:**
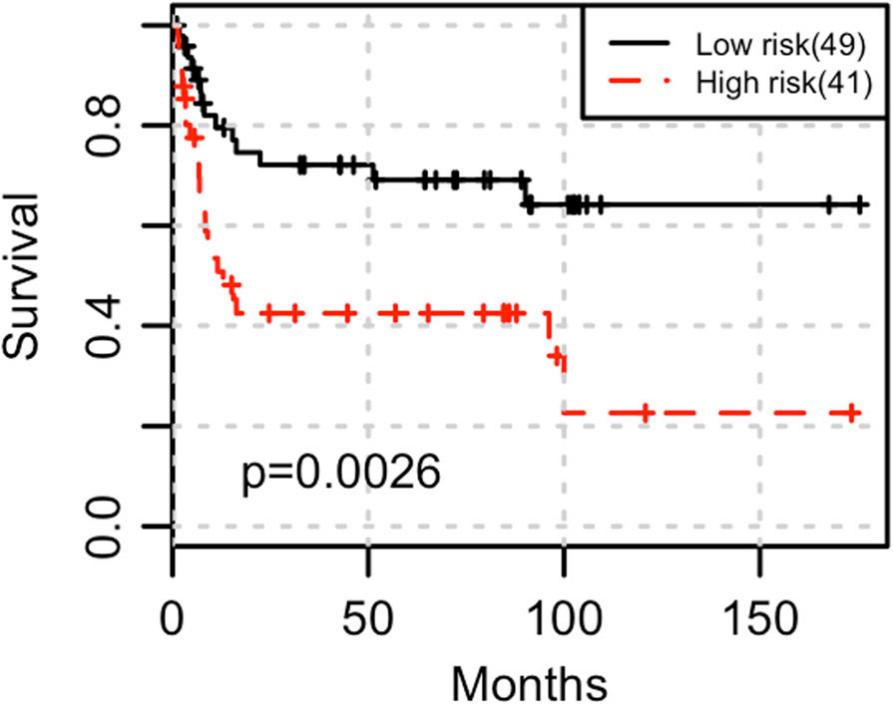
Kaplan-Meier curve of two groups of samples stratified using gene module LRR_2_ in GSE31684. GSE31684 is a bladder cancer dataset

Figure 5 shows that gene co-expression module LRR_2_, which contains 189 genes, is strongly associated with survival (*P* value = 0.00260). Although gene module PCC_2_ identified by PCC based method has substantial overlap with LRR_2_ (PCC_2_ contains 268 genes, where 101 genes are also in LRR_2_), it is not significantly associated with survival (*P* value = 0.200).

### Application to single-cell RNA sequencing data

Single-cell RNA sequencing (scRNA-seq) techniques have provided powerful tools in studying cellular heterogeneity. In this section, we demonstrate that our method can be extended to scRNA-seq data by applying it on a large scRNA-seq dataset of melanoma cells (GSE72056) [28]. GSE72056 contains 1257 malignant cells, 3256 non-malignant cells and 132 unresolved cells with expression profiles of 23,686 genes. Only the malignant cells were used for gene co-expression analysis.

Expression level of gene *i* in cell *j* is quantified as *G*_*ij*_=*log*_2_(TPM_*ij*_/10+1), where TPM_*ij*_ is Transcript-Per-Million (TPM) for gene *i* in cell *j*. In scRNA-seq, dropout event often occurs due to the low number of RNA transcriptomes, which means that expression measurements of some random sampling of transcripts can be missed as zeroes. To account for the dropout events and noise in scRNA-seq data, a different pre-processing method was applied. Firstly, we remove genes with zero expression levels in all cells. Then, we filter out genes with the lowest 80% of mean expression level or genes with the lowest 80% of variance. In GSE72056, 3630 genes were retained after pre-processing.

**Fig. 6:**
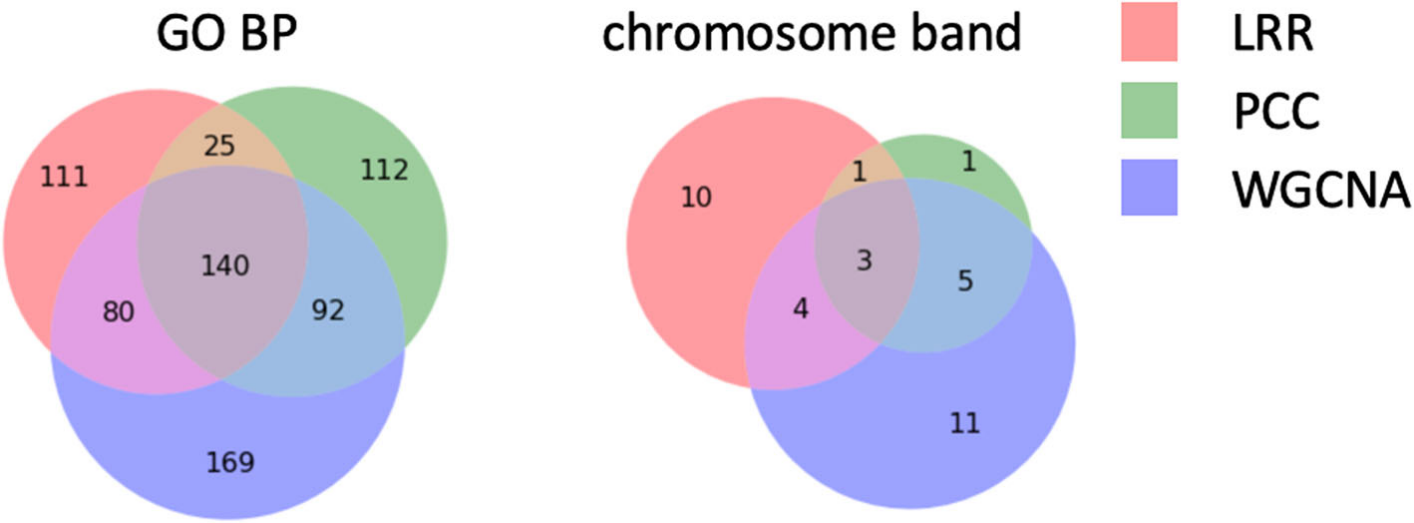
Number of enriched GO BPs and chromosome bands in a melanoma cell dataset GSE72056. *P* value cutoff = 0.01

In total, 18, 11 and 16 gene co-expression modules were identified by our method, PCC based lmQCM and WGCNA respectively. Figure 6 provides the number of enriched GO BPs and chromosome bands with a 0.01 *P* value cutoff. Similar to results in microarray datasets, our method produces results with substantial overlap with existing GCNA methods, while demonstrating the ability to discover new related biological annotations. Table 7 and Table 8 list the most significant GO BPs and chromosome bands that are only enriched when using our method. TCGA skin cutaneous melanoma dataset (363 patients) was used to validate chromosome bands enrichment results. We observe that a large number of patients (3% - 9%) in the TCGA cohort have CNVs in genes on the chromosome bands that are uniquely enriched by our method.

**Table 7:** The most significant GO BPs only enriched using our method in GSE72056

GO ID	Name	*P* value	Module ID
GO:0006259	DNA metabolic process	1.32e-10	6
GO:0006260	DNA replication	2.59e-07	6
GO:0006281	DNA repair	3.17e-06	6
GO:0006261	DNA-dependent DNA replication	5.10e-06	6
GO:0046794	transport of virus	8.60e-06	2

*P* value cutoff = 0.01

**Table 8:** The most significant chromosome bands only enriched using our method in GSE72056

chromosome band	*P* value	Module ID
5p15.33	1.41e-06	18
6p22.1	3.44e-05	15
17q11.2	8.46e-05	11
17q11-q12	1.83e-03	11
6q24.1-q24.2	1.83e-03	11

*P* value cutoff = 0.01

Similar to results in microarray datasets, we also observe that difference in CCI values of gene co-expression modules can contributes to difference in enriched biological annotations and density of PPI networks in scRNA-seq data. For example, module LRR_2_ (112 genes, CCI = 0.282) and module PCC_3_ (95 genes, CCI = 0.324) have large overlap with 45 genes in common. However, GO:0044237 (cellular metabolic process) is more enriched in LRR_2_ (*P* value 5.85e-42 vs. 2.84e-31) and LRR_2_ has higher PPI density (0.0786 vs. 0.0708).

## Discussion

In this paper, we present a new approach for generalizing the traditional correlation-based GCNA methods beyond pairwise relationships between genes. This generalized GCNA method greatly expands the scope for exploring complex relationships among genes. This approach combines a subspace clustering algorithm, LRR, with a weighted network mining algorithm, lmQCM, and makes two major improvements comparing with previous methods. Firstly, comparing with current GCNA algorithms, which mostly calculates the similarity between genes based on pairwise correlation coefficients, we introduce subspace clustering into GCNA to find new biologically meaningful gene modules that can not be characterized based on pairwise relationships. Since the LRR approach does not limit the dimensionality of the subspace, it can accommodate complex relationships which often imply multiple gene or pathway interactions or more variable structural variations. Secondly, comparing with current subspace clustering algorithms in [17, 18], which assign every gene into a cluster, we choose to use lmQCM, which identifies densely connected modules such as quasi-cliques in weighted networks. Our method is more consistent with the fact that some genes could participate in multiple biological processes. In addition, our method can be applied to different kinds of gene expression data, including microarray data and scRNA-seq data.

Despite the advantages demonstrated in this paper, there still exists limitations in our method. As mentioned in [29], when using the data itself as the dictionary, LRR may not be able to exactly recover the subspaces when data contains certain types of errors, such as dense noise. Moreover, a critical issue of LRR is how to estimate or select the parameter *λ*. When data is contaminated by various errors, the selection of *λ* could be quite challenging. We used a cross-validation style approach to overcome this challenge. However, our approach is quite computationally expensive and efficient ways for parameter estimation should be studied in the future.

## Conclusion

In conclusion, we developed a new generalized gene co-expression analysis algorithm based on subspace clustering that works beyond pairwise relationships between genes. Correlations between genes have been shown to be very useful in identifying gene co-expression modules with biological meanings. However, our method provides a complement to existing GCNA methods by using subspace clustering to identify gene co-expression modules with expression profiles of higher intrinsic dimensionalities. We demonstrate that our method can be applied to various types of gene expression data, including microarray data and the emerging scRNA-seq data. By combining our method with other GCNA tools, we can obtain a more comprehensive understanding of gene expression data.

## Abbreviations

ALM: Augmented lagrange multiplier
CCI: Centralized concordance index
CNA: Copy number alternation
CNV: Copy number variation
GCNA: Gene co-expression network analysis
GEO: Gene expression omnibus
GO BP: Gene ontology biological process
lmQCM: Local maximal quasi-clique merger
LRR: Low-rank representation
PCC: Pearson correlation coefficient
PPI: Protein-protein interaction
SCC: Spearman correlation coefficient
scRNA-seq: Single-cell RNA sequencing
SSC: Sparse subspace clustering
TCGA: The cancer genome atlas
TPM: Transcript-per-million
